# *Streptomyces* as Potential Synthetic Polymer Degraders: A Systematic Review

**DOI:** 10.3390/bioengineering8110154

**Published:** 2021-10-23

**Authors:** Maria Fernanda Rodríguez-Fonseca, Jeysson Sánchez-Suárez, Manuel Fernando Valero, Sonia Ruiz-Balaguera, Luis Eduardo Díaz

**Affiliations:** 1 Master in Process Design and Management, School of Engineering, Universidad de La Sabana, Chía 250001, Colombia; mariarofo@unisabana.edu.co; 2 Bioprospecting Research Group, School of Engineering, Universidad de La Sabana, Chía 250001, Colombia; jeyssonsasu@unisabana.edu.co; 3 Energy, Materials and Environment Group, School of Engineering, Universidad de La Sabana, Chía 250001, Colombia; manuel.valero@unisabana.edu.co; 4 Conservation, Bioprospecting and Sustainable Development Group, Environmental Engineering Program, Universidad Nacional Abierta y a Distancia (UNAD), Bogotá 110911, Colombia; sonia.ruiz@unad.edu.co

**Keywords:** biodegradation, *Streptomyces*, polyethylene, polypropylene, plastics, commodity plastics

## Abstract

The inherent resistance of synthetic plastics to degradation has led to an increasing challenge of waste accumulation problem and created a pollution issue that can only be addressed with novel complementary methods such as biodegradation. Since biocontrol is a promising eco-friendly option to address this challenge, the identification of suitable biological agents is a crucial requirement. Among the existing options, organisms of the *Streptomyces* genus have been reported to biodegrade several complex polymeric macromolecules such as chitin, lignin, and cellulose. Therefore, this systematic review aimed to evaluate the potential of *Streptomyces* strains for the biodegradation of synthetic plastics. The results showed that although *Streptomyces* strains are widely distributed in different ecosystems in nature, few studies have explored their capacity as degraders of synthetic polymers. Moreover, most of the research in this field has focused on *Streptomyces* strains with promising biotransforming potential against polyethylene-like polymers. Our findings suggest that this field of study is still in the early stages of development. Moreover, considering the diverse ecological niches associated with *Streptomyces*, these actinobacteria could serve as complementary agents for plastic waste management and thereby enhance carbon cycle dynamics.

## 1. Introduction

Natural and synthetic plastics have replaced numerous materials across industries due to their versatility and overall resistance [1]. These plastics have a desirable strength coupled with outstanding flexibility [1,2]. Both natural and synthetic plastics show high resistance against microbial attacks, but synthetic plastics show better performance over long-term exposure [3], and this higher microbial resistance makes them preferred materials in different industries.

Considering the growing importance of synthetic plastics in our daily lives, their production has risen in the past decade, reaching over 348 million tons worldwide in 2017 [3] and projected to reach 500 million tons by the end of 2020 [4]. From this overall production, five plastics, polyethylene terephthalate (PET), high-density polyethylene (HDPE), polyvinyl chloride (PVC), low-density polyethylene (LDPE), and polypropylene (PP) [5], stand out for their massive scales of production and consumption. However, this increase in production and demand has also led to a rise in waste accumulation, since less than 8 million tons (±2.3% of the global production) of plastic are recycled each year worldwide [3], and these five plastics are especially resistant to environmental degradation.

The growing significance of this plastic waste accumulation is underlined by the negative effects of this waste on human health and the tendency of these materials to persist in the environment for extremely long periods of time [1,2]. In some environments, such as marine systems, plastic pollution can cause physical harm to different animal species [5], with the plastic itself or the byproducts released by the deterioration of the material causing damage to ecosystems and the organisms in it [1,5].

The main concerns associated with plastic waste accumulation are the persistence of this form of waste in every ecosystem and the resultant bioaccumulation of toxic pollutants [6]. Different compounds are released into the environment with the deterioration of plastic materials, including halogenated and aromatic compounds, furans, mercury, brominated compounds, and dioxins [7]. Among these, dioxins are even classified as persistent organic pollutants (POPs) that tend to bioaccumulate and can cause neurological damage, among other health problems [8]. In fact, all of these pollutants have adverse effects on the environment and human health. For instance, they increase air pollution and are linked to problems in the central nervous system, cancer development, and mutations [1,7,9,10].

Micro- and nano-plastics are also a source of considerable concern since the debris of these forms of plastic has been identified in terrestrial and aquatic ecosystems [6,9]. Some studies have shown that 10% of the total global production of plastics ends up in the ocean [11]. This micro- and nano-plastic debris can cause damage to biological species since it is ingested by organisms and causes adverse effects on the organisms’ systems and subsequently bioaccumulates and harms larger organisms [9], thereby affecting the food chain.

Considering the growing scale of this waste management problem, there is an urgent need for the implementation of different methods to control and diminish plastic accumulation in the environment. Because plastic waste is considered hazardous by the Basel Convention, these methods are highly regulated [12]. The byproducts of plastic degradation must be treated as potential hazards as well. The main methods used for the disposal of plastic waste are incineration (pyrolysis), burial or landfilling, and degradation [1]. Incineration and landfilling are the most common methods used at present. In Europe, almost 31% of plastic waste is landfilled, and up to 39% is incinerated as an energy recovery method [13]. However, both methods have disadvantages attributable to the inherent properties of these plastics. Incineration requires high temperatures since the majority of the plastics have substantial thermal resistance [1,13], thereby increasing the energetic demand. Moreover, the incineration of plastics is responsible for the release of toxic compounds into the environment, global warming, and other public health issues [1]. On the other hand, disposal in landfills can cause accumulation of plastic waste underground and the release of leachates with highly toxic compounds as potential groundwater pollutants [1].

To address these problems, degradation methods that “weaken” the polymer chain have been proposed to make the subsequent management of plastic waste more accessible [1]. Thermal treatment, UV irradiation, and chemical or physical treatments can be used to degrade plastics and damage their backbone [1], reducing their resistance to environmental abrasion. One degradation method gaining importance is biodegradation, which uses microorganisms to damage the polymer backbone [14]. Biodegradation presents several advantages over other degradation methods since it has minimal negative effects on the environment [15,16].

Biodegradation, or bioremediation, is a helpful strategy to eliminate pollutants from the environment. Microorganisms or their products (such as enzymes) have been used to degrade a wide variety of substrates, including pesticides, heavy metals, and commodity plastics [15,17,18]. One of the main advantages of biodegradation is its tendency to induce mineralization [1], in which the substrate is “broken” into smaller and simpler molecules used as an energy source by the microorganisms. The byproducts of these reactions are then used to mineralize the environment in which the microorganism is found [1,19]. Mineralization can reduce the deleterious effects of these pollutants on the ecosystems. A crucial point for consideration here is that 100% biodegradation of synthetic substrates is not possible [1], and pretreatment is required to increase the yield of this process [20]. Since synthetic plastics are new materials in the environment, evaluation of microorganisms’ adaptation to these plastics is of importance in determining their ability to degrade such substrates [1].

Bacteria and fungi have been studied for their comprehensive metabolism, which allows them to biodegrade different substrates [21,22,23,24]. As a result of these abilities, the microorganisms themselves can induce deterioration of different substrates and then degrade them with high efficiency [22,24]. Moreover, some research on the metabolites produced by these microorganisms has shown promising results against natural and synthetic plastics [25,26,27]. In such assessments, bacteria are preferred over fungi since their growth is faster [28]. Since plastics are relatively new in the environment, well-known bacteria, such as *Pseudomonas* and *Bacillus*, as well as the fungus *Aspergillus* have been widely studied as possible plastic-degrading microorganisms. Some studies have shown that these microorganisms can cause degradation of several synthetic plastics, such as polyvinyl chloride (PVC) and high-density polyethylene (HDPE) [29,30], with PVC films showing up to 19% weight loss after treatment with the bacterial strains and the HDPE surface showing colonization with dark brown fungus [29,30]. Moreover, *Pseudomonas* strains have been studied for their ability to induce degradation of natural and synthetic rubber (up to 18%), showing the formation of representative degradation byproducts after the incubation time [31].

Another predominant bacterial phylum that is present in several ecosystems and is of increasing importance in the biotechnology field is Actinobacteria. This phylum has been a research target due to its wide and diverse secondary metabolism [32]. Actinobacteria are recognized by their enzyme production and anticancer, antifungal, and antibacterial activities [32]. This phylum has proven to be important for the advancement of pharmaceutical research and biotechnology in general.

*Streptomyces* are one of the main genera of this phylum. These aerobic, Gram-positive bacteria are essential in the biotechnology field since 75% of the commonly used antibiotics are derived from these bacteria, and they produce nearly 5000 of the bioactive compounds reported to date [33,34]. These bacteria have shown great potential in several industries, and their enzyme-production characteristics and the ability to degrade polysaccharides [32] make them an interesting alternative for bioremediation and biological control of a wide range of substrates in different ecosystems.

*Streptomyces* have been studied as bioremediation agents of synthetic [20,35] and natural plastics [36,37]. For synthetic plastics, research has shown promising results for deterioration and weight loss of the initial sample. For natural plastics (e.g., cellulose, lignin, and chitin), these bacteria have been shown to degrade almost all the initial samples [38]. Despite this research attention and their potential capabilities, biodegradation of synthetic plastics with *Streptomyces* has not been studied in detail. However, as stated before, this genus has considerable potential to serve as a solution for the plastic waste accumulation problem. Therefore, this systematic review aimed to identify and evaluate the evidence regarding the potential of *Streptomyces* strains as biodegrading agents for plastic waste.

## 2. Materials and Methods

### 2.1. Search Strategy

For a broad analysis, the search was conducted using the following databases: Scopus, Web of Science, PubMed, and the Google Scholar search engine. The search terms and Boolean operators used were defined as follows:

- Scopus: TITLE-ABS-KEY (streptomyces AND (plastic OR polymer OR polyethylene OR polystyrene OR polypropylene OR polyurethane OR “polyethylene terephthalate” OR “polyvinyl chloride”) AND (degradation OR biodegradation));
- Google Scholar: streptomyces plastic polymer polyethylene polystyrene polypropylene polyurethane “polyethylene terephthalate” “polyvinyl chloride” degradation biodegradation;
- Web of Science/PubMed: (streptomyces AND (plastic OR polymer OR polyethylene OR polystyrene OR polypropylene OR polyurethane OR “polyethylene terephthalate” OR “polyvinyl chloride”) AND (degradation OR biodegradation)).

Figure S1 shows the distribution of the number of hits between the search terms (i.e., *Streptomyces*, plastics-related and degradation-related).

### 2.2. Inclusion and Exclusion Criteria

The following inclusion criteria were used to select the articles: (a) original research articles, (b) studies evaluating the degrading potential of a *Streptomyces* strain, and (c) studies on the degradation of synthetic plastic. The exclusion criteria were as follows: (a) articles written in a language other than English, and (b) articles where the determination method for the degradation was not reported.

To avoid bias in article selection, the selection process was performed separately in a blinded manner by each of the three researchers; thus, each researcher assessed the titles and abstracts individually using the Rayyan QCRI tool [39]. An article was marked as potentially included if two researchers indicated that it met the inclusion/exclusion criteria. If only one of the researchers considered the article to have met the inclusion criteria, a discussion was held to address the discrepancies. The potentially included articles were then assessed at the full-text level. The articles that met the inclusion/exclusion criteria at this point were selected for data analysis.

### 2.3. Data Collection and Tabulation

An acquisition form was designed to ensure careful data collection. The form was designed by one researcher and evaluated by another to avoid collection bias. Once the final version of the form was determined, it was used for data collection from the selected articles. Data were tabulated using the form by one researcher and validated by a second researcher.

## 3. Results

### 3.1. Selection and Characteristics of Studies

**Figure 1:**
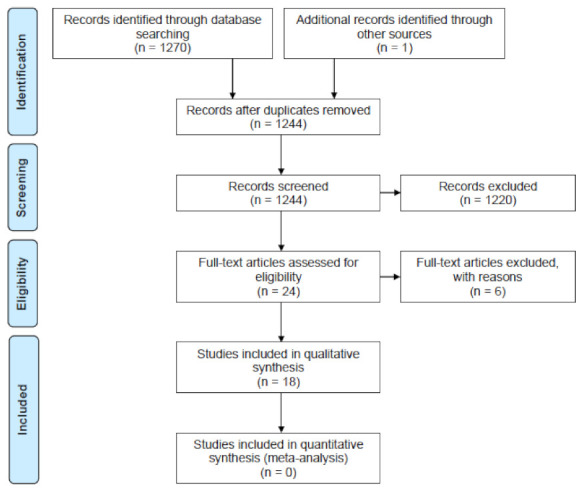
Flowchart of systematic literature search according to PRISMA guidelines. Modified from the work of [40]. The systematic review was done following the PRISMA guidelines, the complete checklist can be reviewed in Table S1.

A total of 1244 non-duplicate articles were identified in the literature search. Since this review was focused on the degradation of synthetic polymers, articles that evaluated the degradation or production of natural polymers were discarded. In the first screening, 1220 studies were excluded because they were not related to synthetic polymer degradation by *Streptomyces* strains, were not original articles, or did not include access to the full text. Of the 24 remaining articles, 18 were selected by full-text screening on the basis of the inclusion/exclusion criteria and were included for data extraction, as shown in Figure 1.

On the first screening, research on biodegradation of synthetic polymers by *Streptomyces* strains was noticeably less developed than other bioremediation research, and almost all of the existing research and development seemed to be focused on the same polymer, polyethylene. Despite the increasing relevance of single-use and non-degradable plastics in the plastic waste problem [41], this field has not been studied in-depth since the main waste pollutants in the published studies were not representative of these plastics.

Additionally, despite the bioremediation and enzyme-production potential of *Streptomyces* and the abundance of this genus in nature, research on synthetic polymer degradation was primarily focused on fungi and other bacteria [22], mainly because of their ability to use almost any complex substrate available in their environment [30].

### 3.2. General Findings

The general findings of this review are summarized in Table 1. As the findings indicate, a variety of synthetic polymers have been studied for biodegradation using *Streptomyces* strains, although most of the research has been focused on polyethylene-like plastics. Moreover, polymer film samples, such as polymers with linear structures, were the most commonly studied due to their availability. The research also showed a large variety of incubation times ranging from 5 to 168 days.

Different methodologies are used to confirm that the polymer was degraded. This review found that studies investigated different outcomes, such as weight loss percentage, decay of the tensile strength, reduction in the elongation percentage, and reduction in the molecular weight, to evaluate polymer degradation, as shown in Table 1. The results varied depending on the method used, and a mixture of methods was often required to validate and confirm the degradation of the polymer.

As shown in Table 1, one of the main methods used for demonstrating the degradation of polymers was the determination of the weight loss percentage. This technique uses gravimetric principles to determine the weight difference of the samples, assuming that this difference was attributable to the degradation of the polymer’s structure. One of the main issues with this method is the high possibility of errors caused by substrate characteristics. For instance, in some studies, the weight difference tended to be higher as the bacteria developed biofilms on the material [31], resulting in increased initial weight and yielding false conclusions. This is the main reason why weight loss percentage cannot be used as a single measurement of degradation. Different authors have recommended using a complementary method (i.e., changes in the physicochemical and mechanical properties) to ensure degradation of the polymer’s backbone [56]. Methods involving assessment of physicochemical and mechanical properties (e.g., changes in molecular weight, particle size, tensile strength, elongation percentage, and thermal stability) may provide an idea of the degradation process but cannot be used alone since other factors (e.g., sterilization, radiation) can affect the polymer’s physicochemical and mechanical properties as well [1]. If the degradation mechanism or the polymer’s degradation is well studied and understood, a complementary method can be used to assess specific characteristics of the tested strain (e.g., clear zone formation on rubber degradation and genome sequencing of polyethylene-biodegrading strains) [31,52]. Since the methods reported by the included studies were widely dissimilar, it was not possible to perform a meta-analysis of the collected results.

### 3.3. Exploration of Plastic Biodegradation by Streptomyces

**Figure 2:**
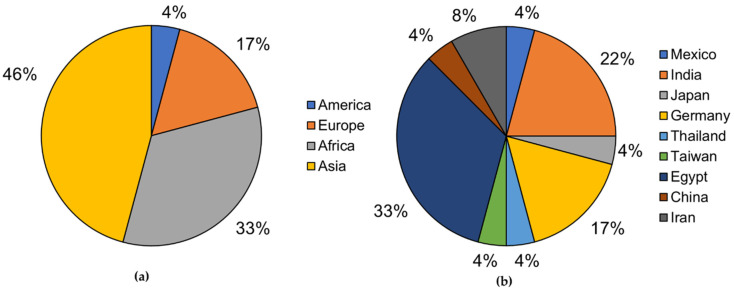
Main isolation countries for *Streptomyces* strains capable of plastic biodegradation: (**a**) Isolated strains’ percentage by continent; (**b**) isolated strains’ percentage by country.

Figure 2 shows that Asia and Africa were the main continents where *Streptomyces* strains with the potential to degrade plastics were isolated (10 and 8 strains, respectively). Egypt and India are the countries leading the search on degrading *Streptomyces*. However, there is no visible trend in the growth of this field since each country has contributed with almost the same amount of research studies.

Notably, none of the studies were conducted in high waste-producing countries or underdeveloped countries with increasing waste management problems. For instance, in Latin America, the development of research focused on the degradation of synthetic polymers is very poor (i.e., 4 papers in 8 years). Despite the fact that Latin America is mostly an underdeveloped region with an increasingly concerning waste problem, no recent studies have been undertaken to identify solutions for this problem [3,4]. This is an unfortunate scenario considering the biodiversity of countries such as Brazil, Mexico, and Colombia [57].

#### Isolation Sources

**Figure 3:**
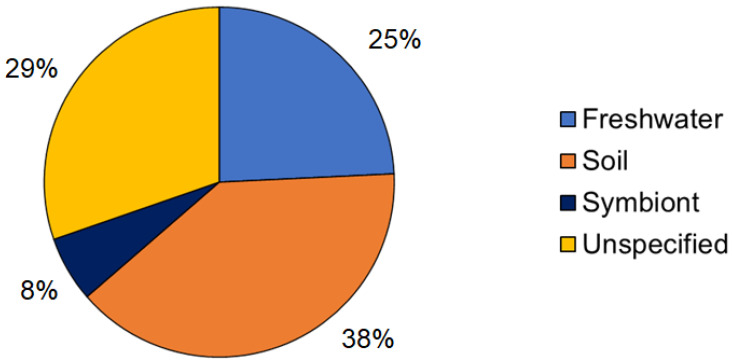
Main isolation sources of *Streptomyces* strains capable of biodegradation of synthetic polymers.

Soil, mainly contaminated soil from landfills or industrial soil, is the main isolation source for bacteria with the potential for degrading synthetic polymers (Figure 3). The exposure to diverse and complex pollutants in such contaminated soil provides selection pressure to promote microorganisms capable of degrading polymers when these polymers are introduced as the sole carbon source [1]. Unpolluted sources were also studied [58], and the organisms isolated from freshwater sources stand out. Thus, *Streptomyces* strains isolated from unpolluted sources may also show the ability to assimilate and degrade complex substrates such as synthetic polymers.

Symbionts from earthworms were also studied [42], and they have shown positive results in the form of changes in the physical properties (e.g., decay on particle size) of low-density polyethylene (LDPE) samples within 4 weeks of incubation. In this study, bacterial strains were isolated from contaminated sources.

One of the major findings of our study is that marine environments have not been explored. Since plastic debris is a major source of contamination in marine ecosystems [11], the potential of marine *Streptomyces* (even other actinobacteria genera) from these environments is an unresolved question with an intriguing research scope [11].

### 3.4. Enzyme Activity

One of the main advantages of microorganisms over other biological sources is their relatively simpler genomics. Elucidation of the molecular mechanisms by which the polymer degradation is achieved can provide an opportunity to improve this capability. Considering the thermodynamic proficiency of enzymes, the identification of enzymes responsible for plastic degradation is the next step after isolation of the microorganisms. For example, hydrolases from fungal and bacterial sources have been employed to biotransform synthetic polymers [59]. However, almost no research has been performed to understand or determine enzymatic activity in these degradation processes or their pathways [60]. Depolymerase-like lipase/esterase, oxygenase, and amylase from bacterial sources have shown positive results in different studies [30,48,52], using techniques such as clear zone formation on agar plates, optimization for enzyme production, specific enzyme activity tests, and whole-genome sequencing. These tests showed the production of enzymes by the *Streptomyces* strains studied, but they did not draw correlations between the production of the enzyme and substrate degradation.

Bode et al., 2001 [31] showed a hypothetical oxidative pathway for the conversion of poly(cis-1,4-isoprene) to acetyl-coenzymeA and propionyl-coenzymeA. Although this was the only study to propose an enzymatic pathway for polymer degradation by *Streptomyces* strains, the authors did not report if the enzyme was being produced by the strains under experimental conditions with the target polymer as a carbon source.

## 4. Discussion

A notable aspect of the literature is the repetition of identified strains across studies since the same species were often studied with the same polymer [45,61], with changes in some experimental variables such as time of exposure. Moreover, some studies have used bacterial consortia to increase the biodegradation rate of xenobiotic pollutants [56], relying on the synergistic activity of multiple strains to degrade the pollutants and their byproducts. However, even though enhanced rates of biodegradation have been reported in these studies, none of these reports described the use of *Streptomyces* consortia.

Despite the well-known ability of *Streptomyces* to degrade several kinds of polymers (e.g., PET, PP, and LDPE) and the significant negative impact of plastic pollution, we found few studies that evaluated *Streptomyces* as a possible agent of biological control. In comparison with other microorganisms such as *Pseudomonas cholororaphis*, *Aspergillus brasiliensis*, and *Chaetomium globosum*, which achieved synthetic plastic biodegradation rates between 9% and 13% [62], *Streptomyces* showed promising activity that deserves further study. Moreover, some studies evaluated the effect of pretreatments on the polymer sample (i.e., grinding the sample, radiation, etc.) [20] and concluded that this technique can enhance the degradation.

Depending on the structure of the sample polymer, measures can be taken to facilitate the process of degradation. For example, higher yields can be obtained with smaller molecules, such as powders, since the superficial area of these materials is higher, better contact is achieved between the microorganisms and the polymer, and the rate of degradation is higher [20]. Moreover, the nature of the sample can have a positive effect on the process and outcomes of degradation; for example, plasticized samples tend to be less degradable since microorganisms degrade plasticizers but not the polymer backbone [63,64].

Another remarkable aspect is the diversity of sources from which the strains in these studies were isolated. Notably, strains isolated from polluted sources (i.e., landfills) tended to have higher yields, which could be attributed to their adaptation to xenobiotic compounds in their native environment [30]. Thus, microorganisms such as bacterial strains from these environments may have hitherto unexplored bioremediation ability [41]. Therefore, marine environments can be an excellent isolation source for further studies and advancements in this field of research.

Considering the diversity of techniques and methodologies used to identify degradation, two or more methods should be used together to obtain more reliable and accurate results. For example, while weight loss on its own can lead to false conclusions and physical changes in the material can be due to external factors [31], a combination of these techniques could more accurately demonstrate the occurrence of plastic degradation.

The primary theory underlying the degradation of these xenobiotic compounds is that they involve enzymatic action since they are complex pollutants [34,65]. One of the insights of this review is that almost no research has been conducted on enzymatic activities or biodegradation pathways, and no genes of specific bacteria have been studied for their role in the degradation of synthetic polymers. These gaps in the existing literature represent promising areas of research since the identification of enzymes or genes that are specifically responsible for polymers’ degradation could facilitate the development of new waste management methods with high yields using *Streptomyces* strains. Sharing the GeneBank access codes of the identified strains is recommended since no phylogenetic analysis could be carried out with the available information. This step can help in further research to identify similar potential strains in the field.

As the metabolic pathways involved in plastic degradation are still underdeveloped, some studies have been focused on understanding and modeling the biodegradation process for selected strains [20,35], aiming to improve the scale-up of these processes and comprehend the bacterial behavior under controlled conditions. These models have been adjusted to first-order kinetic models, showing high adjustment rates on predicting biodegradation of specific polymers by selected strains [20,35], being useful in the design of new bioprocesses at industrial levels, giving solutions on larger scales to the pollution issue regarding plastic waste accumulation. These bioprocesses could lead to a permanent, efficient, and eco-friendly solution, replacing the traditional waste management options available (i.e., incineration, landfills).

## 5. Conclusions

Biological control of plastic pollution represents a promising and eco-friendly management option. In this study, *Streptomyces* strains showed notable potential for degrading synthetic polymers, particularly polyethylene-like polymers. Although the research on biotransformation of single-use plastics is limited, the results obtained so far indicate that studies with this genus are an encouraging research field. On the other hand, despite the widespread use of weight loss as a measure of degradation, it is important to complement weight loss measurements with findings from another method based on a different measurement principle, such as changes in the mechanical properties of the materials or byproduct analysis associated with degradation stages. It is noteworthy that the selected biodegradation or biotransformation approach has to consider the polymer target and the type of sample used (e.g., powder, film, liquid) due to these features represent a methodological challenge for a successful analysis. Certainly, centering the attention on understanding metabolic pathways or kinetic processes could get to a significant breakthrough in implementing these solutions on larger scales. Likewise, enzymatic identification and biochemical characterization, including whole-genome analysis of the known strains, could be helpful in the study of synthetic polymer degraders. We highly recommend and look forward to further research in this area in the upcoming years.

## Figures and Tables

**Table 1:** The characteristics and general specifications of the polymers evaluated in the included studies.

Polymer	Species	Sample Used	Polymer Structure	Time for Degradation	Key Findings	References
Low-Density Polyethylene	*S. fulvissimus*	Compost	Branched ^a^	21 days	Volatile compounds measured ^1^	[42]
	*Streptomyces* sp.	Film	Unspecified ^a^	90 days	5.2% as weight loss percentage ^2^	[43]
	*Streptomyces* sps.	Powder	Branched ^+^	30 to 168 days	46.7% as weight loss percentage	[44]
	*S. badius* (ATCC 39117)	Film	Branched ^+^	15 days	Up to 82% reduction in molecular weight	[45]
	*S. setonii* (ATCC 39116)					
	*S. viridosporus* (ATCC 39115)					
	*Streptomyces* sp.	Film	Linear ^+^	90 days	0.08% as weight loss percentage	[46]
Poly(cis-1,4-isoprene)	*S. griseoplanus* (AS 4.1868T)	Liquid	Unspecified ^a^	70 days	Decay on representative peak ^3^	[47]
	*S. coelicolor* 1A	Film	Branched ^+^	Unspecified	18% as weight loss percentage	[31]
	*S. exfoliatus* (K10)				<3% as weight loss percentage	
	*S. griseus* 1D				18% as weight loss percentage	
	*S. lividans* (1326)				<3% as weight loss percentage	
Polyester-based	*S. antibioticus*	Unspecified	Linear ^+^	5 to 7 days	Clear zone formation and depolymerase production ^4^	[48]
	*Streptomyces* sp.	Powder + Solvent	Linear ^+^	7 days	Clear zone formation	[49]
Polyethylene	*S. aburaviensis*	Film	Unspecified ^a^	7 to 30 days	An average of 28.5% reduction in percent elongation	[50]
	*S. aveblanens*					
	*S. iakyrus*					
	*S. misioensis*					
	*S. warraensis*					
	*S. humidus*					
	*S. nigellus*					
	*S. parvullus*					
	*S. longisporoflavus*	Film	Linear ^+^	60 days	84.02% reduction in tensile strength	[51]
	*S. albogriseolus* (LBX-2)	Film	Unspecified ^a^	15 days	63% reduction in tensile strength	[52]
	*S. badius* (ATCC 39117)	Film	Linear ^+^	30 days	An average of 31% reduction in molecular weight	[53]
	*S. setonii* (ATCC 39116)				An average of 36% reduction in molecular weight	
	*S. viridosporus* (ATCC 39115)				An average of 21% reduction in molecular weight	
	*Streptomyces* sp.	Film	Linear ^+^	Up to 55 days	Up to 12.04% as weight loss percentage	[54]
Polyethylene terephthalate	*Streptomyces* sp.	Powder	Linear ^+^	18 days	Up to 68% as weight loss percentage	[20]
Starch-Polyethylene	*S. badius* (252)	Film	Linear ^+^	20 days	13.6% reduction in tensile strength	[55]
	*S. setonii* (75Vi2)				17.2% reduction in tensile strength	
	*S. viridosporus* (T7A)				12.5% reduction in tensile strength	
High-Density Polyethylene	*Streptomyces*	Powder	Linear ^+^	18 days	Up to 18.26% as weight loss percentage	[35]

^a^ Plastic samples were obtained from industrial soil, and no specific data on raw material were obtained. ^+^ Plastic samples were purchased, and data on raw materials were obtained from suppliers. ^1^ Volatile compounds such as octadecane, eicosane, docosane, and tricosane were identified in treatments containing bacteria. No compounds were found in control treatments without bacteria. ^2^ Weight loss percentage was measured as the difference between the initial and final weights of the sample after the incubation time. ^3^ Decay on the representative peak in HPLC analysis of plastic samples after the incubation time, and samples with no bacterial exposure were used as the negative control. ^4^ Clear zone formation was measured as an “inhibition” zone on agar plate mixed with the targeted polymer; if the strain produced a clear zone, the polymer was capable of poly(cos-1,4-isoprene) degradation.

## Supplementary Materials

The following are available online at https://www.mdpi.com/article/10.3390/bioengineering8110154/s1, Table S1: PRISMA checklist, Figure S1: Venn diagram analysis showing the distribution and relationship between hit results of the search equations.
