# Supplementary material for: Streptomyces as Potential Synthetic Polymer Degraders: A Systematic Review
Source: Bioengineering (Basel). 2021 Oct 23;8(11):154. doi: 10.3390/bioengineering8110154 (PMC8614672; doi:10.3390/bioengineering8110154)
Supplement: Supplementary file 1 [file bioengineering-08-00154-s001.zip › Figure S1.pdf]

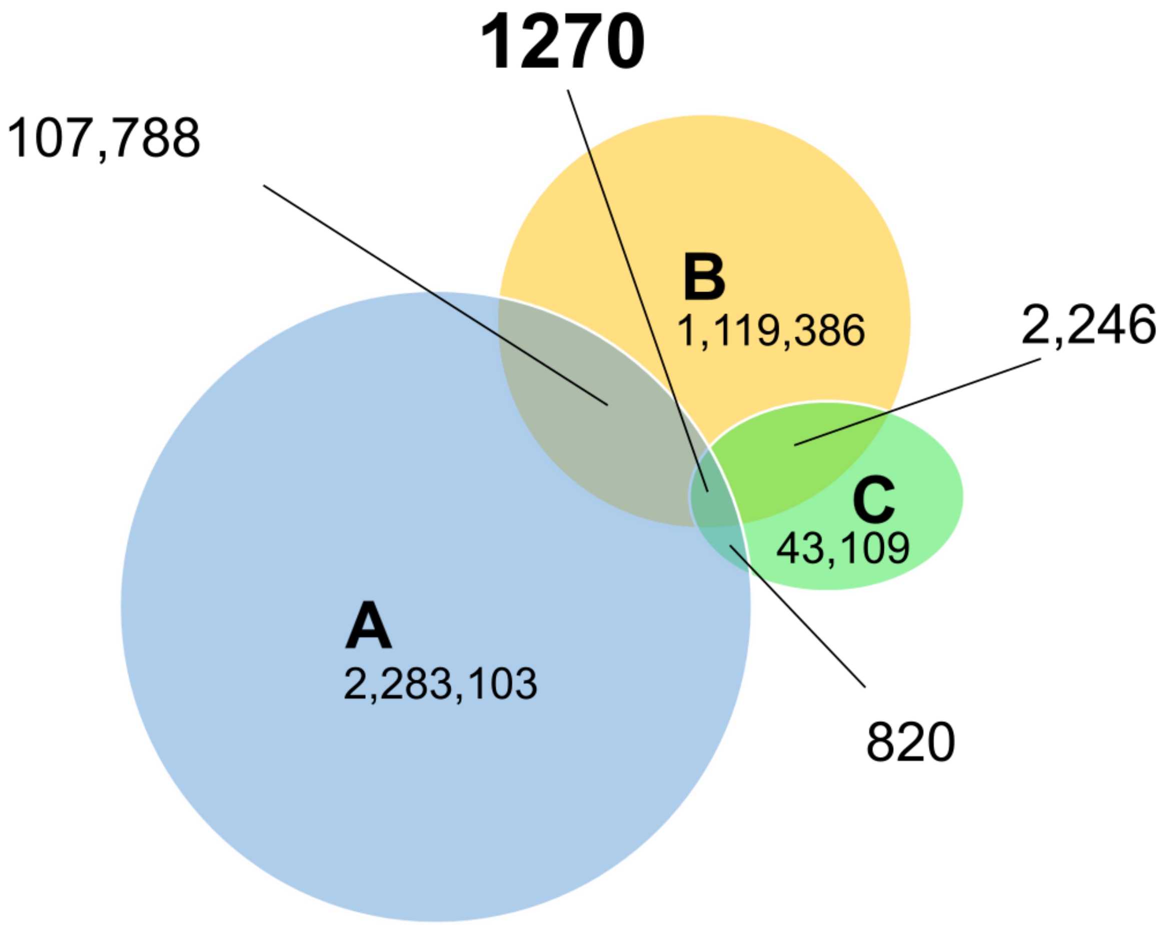

Figure S1. Venn diagram analysis showing the distribution and relationship between hit results of the search equations. Set A is the number of hits of the platics-related terms, Set B is the number of hits of the degradation-related terms and set C is the number of hits of the *Streptomyces* term. The circle sizes is not a scale, their intetion is to provide a visual idea of the differences.
